# Correlations of ICAM-1 gene polymorphisms with susceptibility and multidrug resistance in colorectal cancer in a Chinese population

**DOI:** 10.1097/MD.0000000000007481

**Published:** 2017-08-18

**Authors:** Lu-Bing Liu, Tong Liu, Fu-Ze Xin

**Affiliations:** a Department of Anorectal, Liaocheng People's Hospital, Liaocheng, P.R. China; b Department of Cardiovascular Medicine, Beijing Hospital Affiliated to Peking Union Medical College, Beijing; c Department of Gastrointestinal Surgery, Liaocheng People's Hospital, Liaocheng, P.R. China.

**Keywords:** colorectal cancer, gene polymorphisms, iCAM-1, multidrug resistance, susceptibility

## Abstract

**Background:**

Colorectal cancer (CRC) is a malignant gastrointestinal tumor with a high mortality rate, including both colon and rectal cancer. In order to provide clinical guidance for the treatment of CRC, this study is conducted to investigate the correlations of intercellular adhesion molecule 1 (ICAM-1) gene polymorphisms with susceptibility and multidrug resistance (MDR) of colorectal cancer (CRC).

**Methods:**

A total of 195 patients with CRC were selected as the observation group and 188 healthy people enrolled as the control group. Polymerase chain reaction restriction fragment length polymorphism (PCR-RFLP) was used to test ICAM-1 A13848G and K469E polymorphisms. The expressions of MDR-associated protein topoisomerase II (Topo II) and P-glycoprotein (P-gp) in CRC tissues were detected by immunohistochemistry. The analysis on association of clinical indexes of CRC patients with ICAM-1 gene polymorphisms was performed.

**Results:**

The frequencies of KK genotype and K allele of K469E in the observation group were significantly higher than that in the control group. KE + EE genotype and E allele might be protective factors for CRC. The distribution of genotypes, K469E KK and KE+EE, was highly correlated with histologic grade of tumor differentiation. Compared with adjacent normal tissues, positive rates of Topo II and P-gp expression were significantly increased in CRC tissues. Topo II expression in CRC patients was positively associated with lymph node metastasis and depth of tumor invasion, whereas P-gp expression was only associated with depth of tumor invasion. Higher positive rates of Topo II and P-gp expression were observed in ICAM-1 K469E KK genotype carriers, indicating that ICAM-1 K469E KK genotype might be related to MDR in CRC.

**Conclusion:**

These findings in the current study suggested that ICAM-1 K469E polymorphism is highly correlated with susceptibility and MDR in CRC.

## 1 Introduction

Colorectal cancer (CRC), as a malignant gastrointestinal tumor, includes both colon and rectal cancer. In the recent years, the increasing global incidence and mortality rate of CRC have even surpassed those of gastric and esophageal cancer.^[1]^ Globally, CRC affects 1 million individuals and leads to over 600,000 deaths every year.^[2]^ Patients with diabetes mellitus, smoking, and obesity have been reported to be associated with an increased risk of CRC.^[3]^ CRC is not only correlated to lifestyle but also to genetic factors.^[4]^ Many of localized recurrent CRC tumors adhere to and/or invade to vital pelvic structures, making surgery or radiotherapy palliative. Metastasis is recognized as the most critical concern, which accounts for more than 90% of mortality.^[5,6]^ Multidrug resistance (MDR) is now considered as one of the important factors leading to the failure of chemotherapies and cancer treatment in oncology.^[7,8]^ It consists of several processes, such as, increased drug efflux and decreased accumulation of drugs in cells.^[8]^ As a transporter, MDR-associated protein plays a significant role in MDR by affecting drug transportation of cancer cells and the sensitivity of drugs is related to the MDR1 (a member of MDR family) gene polymorphism.^[9]^

Intercellular adhesion molecule 1 (ICAM-1) plays a vital role in mediating interaction between activated endothelial cells and leukocytes which is responsible for the pathogenesis of atherosclerosis.^[10]^ The ICAM-1 gene positioned on chromosome 19p13.2 has functional activity which has been hinted to exist in the polymorphisms of the ICAM-1 gene.^[11]^ Genetic variations in the ICAM-1 gene can regulate the protein expression and have been extensively investigated in various degenerative and inflammatory diseases.^[12]^ ICAM-1 can be irregularly expressed in CRC and inhibit cancer progression through activation of the host immune monitoring system to prevent cells from leaving the primary tumor, thereby alleviating or eliminating metastasis of CRC.^[13]^ The ICAM-1 gene is made up of 7 exons. The polymorphism of the single nucleotide C to T, located in the sixth exon of the ICAM-1 gene (K469E), contributes to an amino acid substitution (from glutamic acid (E) to lysine (K)) in immunoglobulin-like receptors of the ICAM-1 protein.^[10]^ The E and K alleles are 2 different alleles of the ICAM-1 gene leading to 3 potential genotypes, EE, EK, and KK.^[14]^ A13848G, which also exists in exon 6 of ICAM-1, is found to be related to myocardial infarction.^[15]^

Some polymorphisms are considered as important factors that could contribute to the activity of DNA repair proteins.^[16]^ ICAM-1 K469E polymorphism has been reported that it might be associated with susceptibility to many diseases, such as ischemic stroke (IS), Crohn's disease (CD), coronary heart disease (CHD), and neurocysticercosis (NCC).^[17–20]^ The multidrug resistance (MDR) and expression of P-glycoprotein are the causes of chemotherapy failure. By understanding the mechanism of these causes, they may have clinical significance in overcoming disease caused by drug resistance.^[21]^ Thus, this present study has aimed to examine the associations between K469E polymorphisms of ICAM-1 gene and the susceptibility to CRC and MDR.

## 2 Materials and methods

The Ethics Committee of Liaocheng People's Hospital approved this study. Informed consents from all the patients were signed and obtained. All procedures in the study were in strict accordance with the principles of the Declaration of Helsinki.

### 2.1 Study subjects

A total of 195 patients treated with surgical resection for CRC in Liaocheng People's Hospital between December 2014 and December 2015 were enrolled in this study as the observation group, among which were 102 males and 93 females with a mean age of 56.1 ± 14.2 years. There were 73 patients with colon cancer and 122 patients with rectal cancer, with tumor diameter between 1.75 cm and 9.5 cm. Among 195 patients, 90 patients were accompanied with lymph node metastasis and 105 patients without lymph node metastasis. All the patients were divided into 105 patients with well and moderate differentiation and 90 patients with poor differentiation according to pathological grading of CRC. Based on Duke staging criteria, staging of the patients was done accordingly: A stage—39 patients, B stage—47 patients, C stage—59 patients, and D stage—50 patients.^[22]^ Inclusion criteria for the observational group as follows: all included patients were with first time clinically diagnosed CRC, and the pathological data were complete. Exclusion criteria includes: (1) patients who were postoperatively diagnosed as noncolorectal cancer; (2) patients with other chronic gastrointestinal diseases; (3) patients suffering from acute gastrointestinal disorders within 30 days; (4) patients with incomplete pathological data; (5) patients who had previously been treated with radiotherapy and chemotherapy. Another 188 healthy individuals who took physical examination in Liaocheng People's Hospital were selected as the control group, including 99 males and 89 females with a mean age of 57.2 ± 11.3 years. All research subjects in this study met the inclusion and exclusion criteria.

### 2.2 Sampling

Peripheral venous blood (2 mL) was collected from all subjects (including the observation and control groups) in the morning while they were in a fasting state and was placed in elhylene diamine tetraacetic acid (EDTA) tubes (Beijing Bioteke Corporation, Beijing, China). After fully mixed, the blood samples were cryopreserved in −20°C for DNA extraction.

Tumor tissues and the adjacent normal tissues (over 10 cm away from the tumor tissues) were collected from the observational group (total of 195 patients). All specimens in vitro were collected within duration of 30 minutes and proved pathologically.

### 2.3 Gene polymorphism detection

A13848G and K469E polymorphisms of ICAM 1 gene were detected by polymerase chain reaction restriction fragment length polymorphism (PCR-RFLP). DNA was selected by blood genomic DNA Extraction Kit (Shanghai Biological Engineering Technology & Services Co., Ltd, Shanghai, China). Primers for A13848G and K469E polymorphisms of ICAM-1 gene were designed and synthesized by Shanghai Biological Engineering Technology & Services Co., Ltd (Shanghai, China). After purified and validated by polyacrylamide gel electrophoresis (PAGE), the primer sequences for A13848G and K469E polymorphisms were as follows: A13848G:- forward: 5′-GGAACCCATTGCCCGAGC-3′; reverse: 5′-GGTGAGGATTGCATTAGGTC-3′. K469E:- forward: 5′-CTCCATGTCATCTCATCGTGT-3′; reverse: 5′-CATTATGACTGCGGCTGCTAC-3′.

PCR reaction conditions for A13848G polymorphism were as follows: pre-denaturation at 96°C for 7 minutes, denaturation at 96°C for 30 seconds, anneal at 64°C for 60 seconds, extend at 72°C for 35 seconds, perform a total of 35 cycles, and then extend at 72°C for 7 minutes. PCR reaction conditions for K469E polymorphism were as follows: predenaturation at 95°C for 5 minutes, denaturation at 95°C for 30 seconds, anneal at 60°C for 45 seconds, extend at 72°C for 60 seconds, perform a total of 20 cycles and extend at 72°C for 6 minutes.

PCR reaction products were treated with BstU I restriction enzyme (Beijing Ruida Henghui Biological Technology Co., Ltd, Beijing, China) and Bsh1236I restriction enzyme respectively (Shanghai Biological Engineering Technology & Services Co., Ltd, Shanghai, China) in 37°C water overnight and were analyzed by 2% agarose gel electrophoresis (Shanghai Biological Engineering Technology & Services Co., Ltd, Shanghai, China). Finally electrophoresis results were observed with an ultraviolet gel imager.

### 2.4 Immunohistochemical staining

The biotin-labeled streptavidin peroxidase (SP) method of immunohistochemistry was conducted to determine the expression of MDR-related protein topoisomerase II (Topo II) and P-glycoprotein (P-gp) in CRC patients of the observational group (195 patients).

Initially, all specimens were sliced into 4-μm-thick serial sections and then placed in the 60°C oven for duration of 1 hour. The 4-μm-thick paraffin sections were dewaxed and debenzolized in distilled water, and then immersed into citrate buffer (0.01 M, pH 6.0) with antigen retrieval after inactivation of endogenous peroxidase. The binding activity of nonspecific antigen in the tissue was blocked by adding goat serum and incubating for half an hour in a 37°C thermostat.

JSB-1 and SWT3D1 (mouse anti-human monoclonal antibodies, Shanghai Jinmai Biotechnology Co., Ltd, Shanghai, China) were used as the primary antibodies of Topo II and P-gp, respectively. After incubated with JSB-1 and SWT3D1 at 4°C overnight, the sections were further incubated with secondary antibodies in a 37°C thermostat for 30 minutes. With excess fluid dumped, the third antibodies were added and the tissues were incubated in a 37°C thermostat for 30 minutes. After each time of incubation, the sections were washed thrice with 0.01 M phosphate buffer saline (PBS) for 5 minutes (per each wash) developed in diaminobenzidine (DAB) for 2 minutes and the reaction was stopped by washing with tap water. The sections were counterstained by hematoxylin, then dehydrated and cleared by gradient alcohol and xylene, and finally sealed with neutral balsam.

Topo II was mainly expressed in the nucleus with an even distribution of yellow-brown or yellow particles in the nucleus seen under light microscope, which represents as positive indicator. P-gp was mainly expressed in the membrane with an even distribution of yellow-brown or yellow particles within the membrane or in the cytoplasm as observed under light microscope, which was defined as positive. According to semiquantitative analysis, 10 fields of view (× 400) were selected to calculate percentage of positive cells, on the basis of which scores were given: < 25%, 1 point; 25% to 50% (25% included), 2 points; 50% to 75% (50% included), 3 points; ≥ 75%, 4 points. Staining intensity of positive cancer cells was calculated as following: pale yellow, 1 point; yellow, 2 points; yellow-brown, 3 points. The final score of the field was worked out by multiplying the 2 scores above. The final score of the field was measured as: 0 to 4 point (s), negative (−); 4 to 8 points, positive (+); 8 to 12 points, positive (++).

### 2.5 Statistical analysis

The collected data in the study were analyzed by SPSS 21.0 statistical software (SPSS Inc., Chicago, IL). The representativeness of the samples was tested by Hardy-Weinberg equilibrium (HWE). Enumeration data were tested by using the chi-square test and represented as percentage or rate. Measurement data were represented as mean ± standard deviation. Comparison between groups was calculated by *t*-test. Differences were considered statistically significant with a *P* value < .05.

## 3 Results

### 3.1 Electrophoresis results of ICAM-1 gene polymorphisms

**Figure 1:**
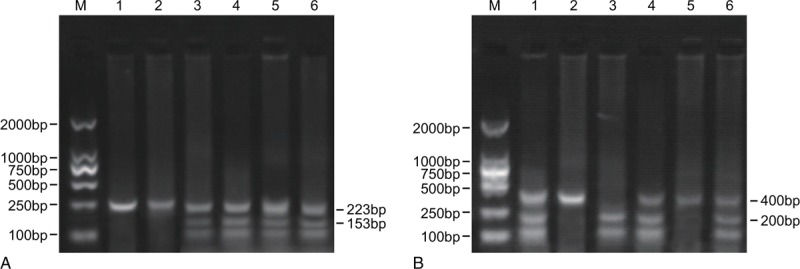
Electrophoresis map of A13848G and K469E polymorphisms. Note: (A) A13848G polymorphism, 1 and 2 represent AA genotype; 3 and 4 represent AG genotype; 5 and 6 represent GG genotype. (B) K469E polymorphism, 1 and 4 and 6 represent KE genotype; 2 and 5 represent KK genotype; 3 represents EE genotype.

As shown in Fig. 1, the amplification length of A13848G gene PCR product was 223 bp, and it could be digested into AA, AG, and GG genotypes by the restriction enzyme. The amplification length of K469E gene PCR product was 153 bp, and it could be digested into KE, KK, and EE genotypes with the help of restriction enzyme.

### 3.2 Distribution of genotype and allele frequency of ICAM-1 gene polymorphisms

Distribution of genotype and allele frequency of ICAM-1 A13848G and K469E polymorphisms in the observation and control groups were tested by using HWE (Hardy-Weinberg equilibrium), and the results showed that the population was well represented by our samples. Our data proved that the distributions of K469E KE + EE genotype and E allele in the control group were significantly higher than that in the observation group (both *P* < .05), indicating that KE + EE genotype and E allele were protective factors for CRC (odds ratio [OR] = 0.574, 95% confidence interval [95%CI] = 0.383–0.861, *P* = .007, OR = 0.639, 95%CI = 0.461–0.886, *P* = .007). No significant difference was found in the distributions of A13848G polymorphism between the observation and control groups (all *P* > .05) (Table 1).

**Table 1:**
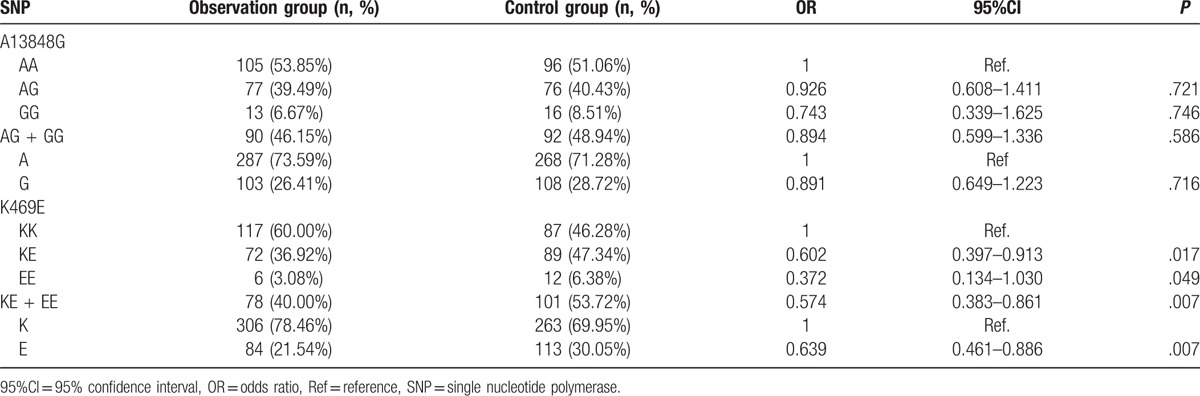
Distribution of genotype and allele frequency of A13848G and K469E polymorphisms in the observation and control groups.

### 3.3 Correlation of ICAM-1 gene polymorphism and clinicopathological factors in CRC patients

As shown in Table 2, the distribution of K469E KK genotype in well- or moderately-differentiated individuals was significantly lower than that in poorly-differentiated CRC individuals. Concurrently, the distribution of KE + EE genotype in well- or moderately-differentiated individuals was significantly higher than that in poorly differentiated CRC individuals (*P* < .05). Correlations of K469E polymorphism with other indexes and correlations of A13848G polymorphism with all indexes in CRC patients were not found in this study (all *P* > .05).

**Table 2:**
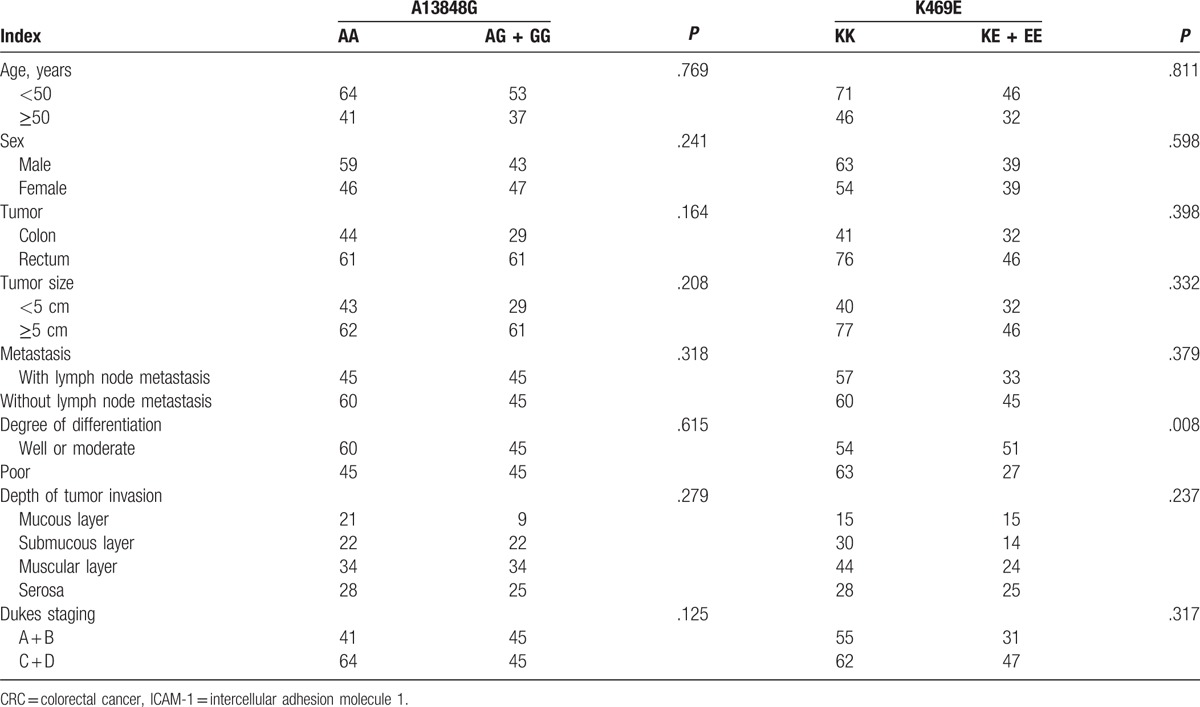
Correlation of ICAM-1 gene polymorphisms with clinical indexes of CRC patients.

### 3.4 Expressions of MDR-associated protein Topo II and P-gp between CRC tissues and adjacent normal tissues

**Figure 2:**
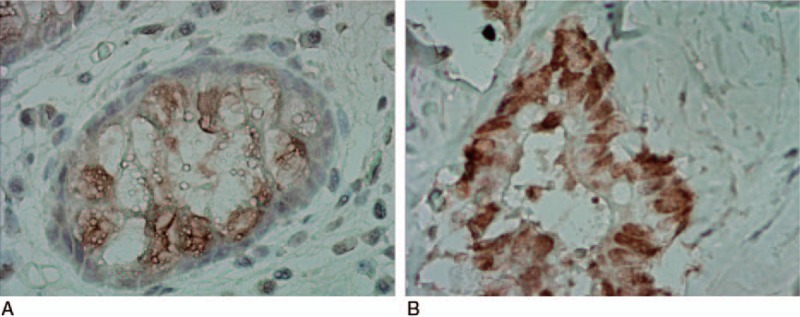
Expressions of MDR-associated proteins (Topo II and P-gp) in CRC tissues. Note: (A) Topo II is mainly expressed in nucleus; (B) P-gP is mainly expressed in cytoplasm and cell membrane. CRC = colorectal cancer, MDR = multidrug resistance, P-gp = P-glycoprotein, Topo II = topoisomerase II.

As shown in Fig. 2, Topo II is mainly expressed in the nucleus, whereas P-gP is mainly expressed in cytoplasm and cell membrane. In the observation group, the positive rate of Topo II and P-gp in CRC tissues were 52.82% and 55.90%, respectively, which were significantly higher when compared to adjacent normal tissues (42.02% and 42.30%) (*P* < .05, Table 3).

**Table 3:**
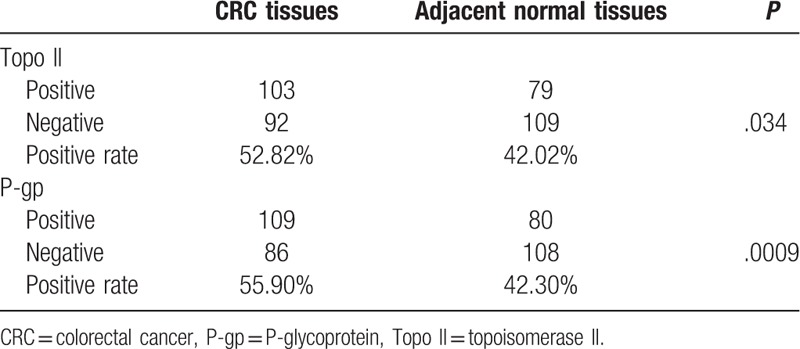
Comparison of Topo II and P-gp expressions between CRC tissues and adjacent normal tissues.

### 3.5 Correlation of expressions of MDR-associated protein, Topo II and P-gp in CRC tissues, and clinical indexes of patients

As shown in Table 4, Topo II expression in CRC tissue was associated with lymph node metastasis and depth of tumor invasion in CRC patients. The positive expression of Topo II in patients with lymph node metastasis was significantly higher than that in patients without lymph node metastasis. Meanwhile, the positive expressions of Topo II in the muscular layer and serosa were also significantly higher than that in the mucous layer and the sub-mucous layer (all *P* < .05). The expression of P-gp in CRC tissues was associated with the depth of tumor invasion in CRC patients and its expressions in muscular layer and serosa which were resulted as significantly higher than that in the mucous layer and the sub-mucous layer (all *P* < .05). No correlations of expressions of Topo II and P-gp in CRC tissues with other clinical indexes of patients were found in the present study (all *P* > .05).

**Table 4:**
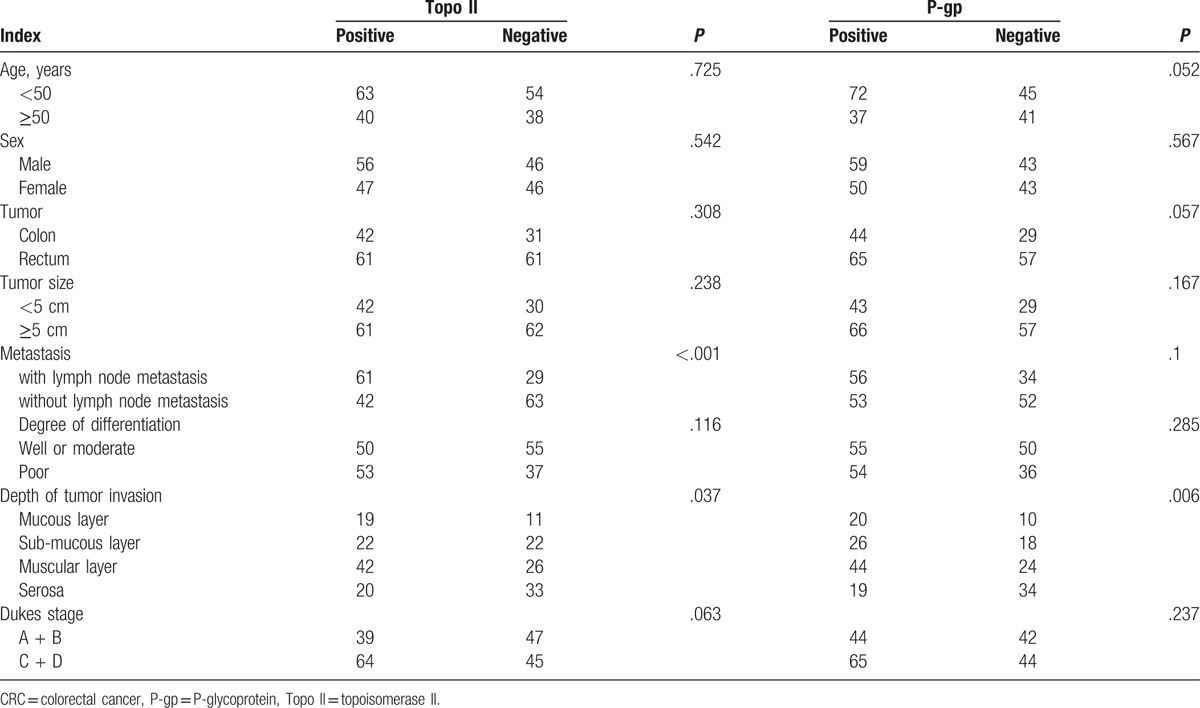
Correlation of Topo II and P-gp expressions in CRC and clinical indexes of CRC patients.

### 3.6 Correlation of expressions of MDR-associated protein Topo II and P-gp in CRC tissues with ICAM-1 genotypes

As shown in Table 5, positive expressions of Topo II and P-gp in CRC patients with K469E KK genotype were significantly higher than that in patients with other genotypes (all *P* < .05), indicating that ICAM-1 K469E KK genotype might be correlated with MDR in CRC. Association between expressions of Topo II and P-gp and CRC patients with different genotypes in A13848G polymorphism of ICAM-1 gene were not at all found (all *P* > .05).

**Table 5:**
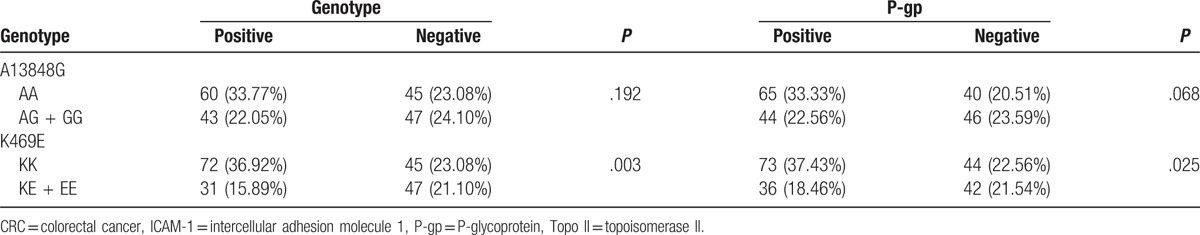
Correlation of Topo II and P-gp expressions in CRC and ICAM-1 genotypes.

## 4 Discussion

The present study interrogated the association between the ICAM-1 gene polymorphism, susceptibility and MDR in CRC. Interestingly, 2 special SNPs, A13848G and K469E in the ICAM-1 gene were discovered. The study proved that K469E gene polymorphisms were associated with susceptibility and MDR in CRC.

The HWE plays a critical role in the investigation of population genetics and provides the sampling distribution of genotype that counts in a sample size with random mating, no mutation, no choice, no migration, and no genetic drift of individuals drawn from a population.^[23]^ Consequently, it was shown that the distributions of polymorphism in A13848G gene and K469E gene were consistent with the HWE and the group representativeness of the sample. Surprisingly, we found that the distributions of the K469E KE + EE genotype and E allele were significantly higher in CRC patients compared with normal individuals. Thus, K469E KE + EE genotype and E allele in ICAM-1 gene might be protective factors for CRC. Wang et al^ [13]^ found that K469E gene polymorphisms were associated with differentiation of CRC because irregular ICAM-1 expression in CRC patients could suppress cancer progression, and these findings may help to evaluate the prognosis of CRC according to the individual differences.

Comparing the relationship between different genotypes of ICAM-1 gene and the clinical parameters (age, sex, tumor location, tumor size, depth of tumor invasion, migration, degree of differentiation, and Dukes staging) of CRC patients, we found that the distribution of K469E KK genotype in poorly differentiated individuals was significantly higher than that in moderately- or well-differentiated individuals (*P* < .05), whereas the distribution of KE + EE genotype in poorly-differentiated individuals was lower than that in moderately- or well-differentiated individuals (*P* > .05). Traditionally, CRC is graded as well-, moderately- or well-differentiated and undifferentiated based upon the foundation of the percentage of gland formation.^[24]^ A recent research study conducted by Kim et al^[25]^ showed that the frequency of poor-differentiated colorectal adenocarcinoma (CRAC) ranging from 3.3% to 18% of all CRCs was low, but its prognosis was reported to be typically poor and more unfavorable than that of moderately- or well-differentiated CRACs.

P-gp DNA Topo II is a good target of anticancer drugs in clinical classes because of the mechanism of specific Topo II inhibitor which reversibly traps the enzyme–DNA complex, thereby turning the Topo to be physiologically toxic, capable of producing permanent DNA damage and leading to cell death.^[26]^ The over-expression of P-gp is a product of multidrug resistant gene 1 and the role of P-gp in drug efflux has been well studied.^[27]^ Our results unveiled that the positive rates of Topo II and P-gp in CRC tissues were 52.82% and 55.90%, respectively, which were higher than those in adjacent normal tissues (42.02% for Topo II, 42.30% for P-gp). Furthermore, we found that expression of Topo II in CRC was associated with lymph node metastasis of CRC patients. The positive expression rate of Topo II in patients with lymph node metastasis was significantly higher than that in patients without lymph node metastasis (*P* < .05). Topo II is a universal nuclear enzyme associated with the control of DNA topology.^[28]^ Topo-II expression reflects the sensitivity of anticancer drugs. When Topo-II expression is high, Topo-II inhibitors are applicable in tumor chemotherapy and will subsequently enhance the curative effect but in the case of reduced Topo II expression, such inhibitors become ineffective because the tumor is insensitive to them.^[29]^ Consequently, we may assume that the expression of Topo II and P-gp in CRC was related to the depth of tumor invasion in CRC patients. A recent study consistent with our assumption demonstrated that the invasive depth of early CRC was fundamentally evaluated by colonoscopic examination combined with chromoendoscopy.^[30]^ We also found that the expressions of Topo II and P-gp in the muscular layer and the serous layer were significantly higher than that in the mucosal layer and the submucosal layer (*P* < .05). Chen et al^[31]^ found that P-gp and Topo II played critical roles in MDR and the detection of expressions of P-gp and Topo II presented a vital guiding significance in chemotherapy for CRC.

Another important finding was that ICAM-1 K469E KK genotype might be associated with MDR of CRC. The positive rates of Topo II and P-gp expression were significantly higher in the patients with ICAM-1 K469E KK genotype than those with other genotypes. MDR-associated protein was related to individual differences and gene transfection study which suggested that MDR-associated protein may confer drug resistance by decreasing drug accumulation in the cells.^[32]^ ICAM-1 provides critical adhesion molecules for macrophages (MF)-mediated drug resistance and it attenuates MF-mediated multiple myeloma cell drug resistance.^[33]^ ICAM-1 gene polymorphism at K469E may have an effect on the plasmas ICAM-1 expression.^[34]^ Therefore, ICAM-1 gene polymorphisms at K469E may be related to the MDR of CRC.

## 5 Conclusion

In conclusion, our collected data demonstrated that the ICAM-1 K469E polymorphism was associated with the susceptibility and MDR in CRC in a Chinese population. Although there were some existing limitations in the study regarding the diversity or ethnicity of samples, our findings successfully presented further insights on pathogenesis of CRC and provided reference for clinical diagnosis and treatment of CRC.

## Acknowledgments

The authors would like to acknowledge the helpful comments on this paper received from our reviewers.
